# Characterization of Lgr5+ Progenitor Cell Transcriptomes after Neomycin Injury in the Neonatal Mouse Cochlea

**DOI:** 10.3389/fnmol.2017.00213

**Published:** 2017-07-04

**Authors:** Shasha Zhang, Yuan Zhang, Pengfei Yu, Yao Hu, Han Zhou, Lingna Guo, Xiaochen Xu, Xiaocheng Zhu, Muhammad Waqas, Jieyu Qi, Xiaoli Zhang, Yan Liu, Fangyi Chen, Mingliang Tang, Xiaoyun Qian, Haibo Shi, Xia Gao, Renjie Chai

**Affiliations:** ^1^Key Laboratory for Developmental Genes and Human Disease, Ministry of Education, Institute of Life Sciences, Southeast UniversityNanjing, China; ^2^Research Institute of OtolaryngologyNanjing, China; ^3^Co-innovation Center of Neuroregeneration, Nantong UniversityNantong, China; ^4^Bioinformatics Department, Admera Health LLCSouth Plainfield, NJ, United States; ^5^School of Pharmacy, Institute for Stem Cell and Neural Regeneration, Nanjing Medical UniversityNanjing, China; ^6^Jiangsu Provincial Key Medical Discipline (Laboratory), Department of Otolaryngology Head and Neck Surgery, Affiliated Drum Tower Hospital of Nanjing University Medical SchoolNanjing, China; ^7^Department of Biotechnology, Federal Urdu University of Arts, Science and TechnologyKarachi, Pakistan; ^8^Department of Biomedical Engineering, Southern University of Science and TechnologyShenzhen, China; ^9^Department of Otorhinolaryngology Head and Neck Surgery, The Sixth People's Hospital Affiliated to Shanghai Jiao Tong UniversityShanghai, China

**Keywords:** neomycin, hair cell injury, mRNA-Seq, hair cell regeneration, supporting cell, proliferation, differentiation, gene expression

## Abstract

Lgr5+ supporting cells (SCs) are enriched hair cell (HC) progenitors in the cochlea. Both *in vitro* and *in vivo* studies have shown that HC injury can spontaneously activate Lgr5+ progenitors to regenerate HCs in the neonatal mouse cochlea. Promoting HC regeneration requires the understanding of the mechanism of HC regeneration, and this requires knowledge of the key genes involved in HC injury-induced self-repair responses that promote the proliferation and differentiation of Lgr5+ progenitors. Here, as expected, we found that neomycin-treated Lgr5+ progenitors (NLPs) had significantly greater HC regeneration ability, and greater but not significant proliferation ability compared to untreated Lgr5+ progenitors (ULPs) in response to neomycin exposure. Next, we used RNA-seq analysis to determine the differences in the gene-expression profiles between the transcriptomes of NLPs and ULPs from the neonatal mouse cochlea. We first analyzed the genes that were enriched and differentially expressed in NLPs and ULPs and then analyzed the cell cycle genes, the transcription factors, and the signaling pathway genes that might regulate the proliferation and differentiation of Lgr5+ progenitors. We found 9 cell cycle genes, 88 transcription factors, 8 microRNAs, and 16 cell-signaling pathway genes that were significantly upregulated or downregulated after neomycin injury in NLPs. Lastly, we constructed a protein-protein interaction network to show the interaction and connections of genes that are differentially expressed in NLPs and ULPs. This study has identified the genes that might regulate the proliferation and HC regeneration of Lgr5+ progenitors after neomycin injury, and investigations into the roles and mechanisms of these genes in the cochlea should be performed in the future to identify potential therapeutic targets for HC regeneration.

## Introduction

Sensory hair cells (HCs) in the inner ear are specialized mechanoreceptors for sound recognition. Damage to these HCs leads to sensorineural hearing loss, which is one of the most common sensory disorders affecting millions of children and adults around the world (Duthey, 2013). HC damage is irreparable in adult mammals due to the absence of cochlear HC regeneration, and this results in permanent hearing loss. In contrast, the supporting cells (SCs) located within the auditory and vestibular system of non-mammalian animals such as birds and fish retain the ability to proliferate and regenerate HCs in response to both internal and external auditory/vestibular damage (Corwin and Cotanche, 1988; Balak et al., 1990; Stone and Cotanche, 2007; Ma et al., 2008; Warchol, 2011). Several recent studies have shown that a subset of SCs expressing Lgr5 can act as progenitor cells in the mammalian cochlea, and these cells possess limited regenerative capacity during the early postnatal period (Bermingham-McDonogh and Reh, 2011; Chai et al., 2011, 2012; Shi et al., 2013; Bramhall et al., 2014; Cox et al., 2014; Waqas et al., 2016a). HC injury in the neonatal mouse cochlea can initiate self-repair processes that involve the spontaneous regeneration of HCs from these Lgr5+ progenitors, and this has been demonstrated both *in vitro* and *in vivo* (Bramhall et al., 2014; Cox et al., 2014). However, this regenerative ability is lost as the mice age and disappears completely by the time they reach adulthood (White et al., 2006; Oesterle et al., 2008; Cox et al., 2014).

In the organ of Corti, the specific arrangement of SCs and sensory HCs is not only necessary to maintain the mosaic-like structure, but the SCs might also serve as a reservoir for regenerating HCs after damage (Li et al., 2003; Lee et al., 2006; Sinkkonen et al., 2011; Cox et al., 2014; Li W. et al., 2015). Although the resident SCs in the cochlea are postmitotic by nature or due to the complex organization of the organ of Corti (Malgrange et al., 2002; Waqas et al., 2016b), these SCs can be cultivated *in vitro* and have been shown to form floating spheres with the ability to differentiate into various cell types of the inner ear, including HCs (Oshima et al., 2007a; Martinez-Monedero et al., 2008; Wang T. et al., 2015). *In vitro* and *in vivo* regulation of key developmental factors such as Wnt (Malgrange et al., 2002; Yamamoto et al., 2006; Shi et al., 2013; Liu L. et al., 2016), Notch (Li et al., 2003; Doetzlhofer et al., 2009; Kelly et al., 2012; Ni et al., 2016), and Atoh1 (Zheng and Gao, 2000; Shi et al., 2012; Kuo et al., 2015) in these SCs can stimulate the increased formation of myosin7a+ HCs. In addition, studies have shown that upon cochlear HC damage, non-sensory SCs/progenitors display at least some capacity to proliferate and mitotically regenerate HCs as a self-repair response (Li et al., 2003; Cox et al., 2014). To better understand the HC regeneration mechanism and to develop strategies to promote HC regeneration in adult mammals, it is important to identify the key genes involved in the HC injury-induced self-repair response, including proliferation of SCs/progenitors and their differentiation into HCs.

Lgr5 is a downstream target gene of the Wnt pathway and is a marker for adult stem cells that is expressed in a subpopulation of cochlear SCs (Chai et al., 2011). In the inner ear, Lgr5+ progenitors exist in a quiescent state, but they have been shown to proliferate and regenerate HCs via both mitotic division and direct transdifferentiation after HC injury (Madisen et al., 2010; Chai et al., 2012; Bramhall et al., 2014; Cox et al., 2014). Genetic ablation of HCs *in vivo* stimulates the Lgr5+ progenitors to acquire the HC fate in all three cochlear turns but with significantly higher frequency in the apex compared to the base (Cox et al., 2014). Similarly, in the *in vitro* ototoxic damage model, the new HCs originate from the Lgr5+ progenitors that are present in the organotypic culture of the neonatal cochlea (Bramhall et al., 2014). These studies have demonstrated that damage to the neonatal cochlea results in regeneration of HCs initiated by the Lgr5+ progenitors. Our previous work also demonstrated that after neomycin injury the Wnt signaling pathway is activated in the cochlea as part of the repair process (Kelly et al., 2012), but the key genes involved in neomycin injury-induced self-repair responses have not yet been identified. It is important to understand the detailed molecular mechanism regulating the ability of Lgr5+ progenitor cells to proliferate and regenerate HCs after neomycin injury because this might provide new targets for stimulating these Lgr5+ progenitors to regenerate more HCs after ototoxic damage and to restore hearing.

In this study, we explored the molecular mechanism behind the proliferation and HC regeneration capacity of Lgr5+ progenitors after neomycin damage. We found that after neomycin treatment, Lgr5+ progenitors located within the neonatal cochlea showed a significantly greater ability to proliferate and regenerate HCs. We further performed RNA-seq profiling of the Lgr5+ progenitors in order to determine the genes involved in regulating proliferation and HC regeneration after neomycin treatment. Finally, we predicted the function of the differentially expressed genes involved in inner ear HC regeneration using the STRING bioinformatics tool to construct a protein-protein interaction network. These datasets are expected to systematically explain the detailed regulatory mechanisms of Lgr5+ progenitors in HC regeneration after neomycin damage.

## Materials and methods

### Animals and genotyping PCR

Lgr5-EGFP-IRES-creERT2 mice (Stock #008875, Jackson Laboratory) and Rosa26-tdTomato reporter mice (Stock #007914, Jackson Laboratory) of either sex were used in the experiments (Pannier et al., 2009). We performed all animal procedures according to protocols that were approved by the Animal Care and Use Committee of Southeast University and were consistent with the National Institute of Health's Guide for the Care and Use of Laboratory Animals. We made all efforts to minimize the number of animals used and to prevent their suffering.

The tail tips were collected from transgenic mice, and genomic DNA was obtained by adding 180 μl 50 mM NaOH, incubating at 98°C for 60 min, and adding 20 μl 1M Tris-HCl (PH 7.0). The genotyping PCR was carried out by using 2 × Tag Master Mix (Vazyme), and the PCR protocol was as follows: 94°C for 3 min; 37 cycles of 94°C for 30 s, 60°C for 30 s, and 72°C for 45 s; 72°C for 5 min; and holding at 4°C. The genotyping primers were as follows: Lgr5 (F) CTG CTC TCT GCT CCC AGT CT, wild-type (R) ATA CCC CAT CCC TTT TGA GC, mutant (R) GAA CTT CAG GGT CAG CTT GC; tdTomato wild-type (F) AAG GGA GCT GCA GTG GAG T, (R) CCG AAA ATC TGT GGG AAG TC; mutant (F) GGC ATT AAA GCA GCG TAT C, (R) CTG TTC CTG TAC GGC ATG G.

### *In vitro* lineage tracing of Lgr5+ cells in the neomycin-damaged and undamaged cochleae

Heterozygous Lgr5-EGFP-creERT2 mice were crossed with homozygous Rosa26-tdTomato mice to trace the fate of Lgr5+ cells in the neomycin-damaged and undamaged cochleae. Postnatal day (P)1 mice were sacrificed, and the cochleae from Lgr5-EGFP-creER/Rosa26-tdTomato double-positive mice were dissected out and cultured in DMEM/F12 medium supplemented with N2 (1:100 dilution, Invitrogen), B27 (1:50 dilution, Invitrogen), heparin sulfate (50 ng/ml, Sigma), and the growth factors bFGF (10 ng/ml, Sigma), EGF (20 ng/ml, Sigma), and IGF-1 (50 ng/ml, Sigma) (full medium). The cochleae were treated with 500 nM 4OH-tamoxifen for 4 days all through the culture. At 12 h after the beginning of the culture, the cochleae were treated with 0.5 mM neomycin (Sigma) or PBS for 12 h. EdU was added to the medium at a final concentration of 10 μM to label dividing cells. The damaged and undamaged cochleae were examined after 4 days of culture.

### Isolation of Lgr5+ cells via flow cytometry

Approximately 30–40 postnatal day (P)1–2 Lgr5-EGFP-creERT2 mice were sacrificed, and the cochleae were dissected out and cultured in full medium as described above and allowed to recover for a few hours. The cochleae were treated with 0.5 mM neomycin (Sigma) or PBS for 12 h and then allowed to recover in full medium for 24 h. The cochleae were collected and trypsinized by prewarmed 0.125% trypsin/EDTA (Invitrogen) at 37°C for 8 min. The same amount of soybean trypsin inhibitor (10 mg/ml, Worthington Biochem) was then added to terminate the trypsin reaction in the neomycin-damaged and undamaged cochlear samples. Cochleae were separated into single cells by pipetting up and down 80–100 times with blunt tips and then percolating through a 40 μm cell strainer (BD Biosciences). Dissociated cells from damaged and undamaged cochleae were sorted on a BD FACS Aria III using the GFP channel.

### Real-time PCR

Total RNA was extracted from ~20,000 FACS-sorted neomycin-treated Lgr5+ progenitors (NLPs) and 20,000 untreated Lgr5+ progenitors (ULPs) with an RNeasy micro kit (QIAGEN). RevertAid First Strand cDNA Synthesis Kit (Thermo) was used to synthesize cDNA. Real-time PCR was carried out by using the SYBR Green PCR Master Mix (Roche) on a BIO-RAD C1000 Touch thermal cycler (BIO-RAD). Each 25 μL PCR reaction mixture contained 12.5 μL 2 × SYBR Green PCR Master Mix, 0.5 μL forward primer (10 μM), 0.5 μL reverse primer (10 μM), 2 μL template, and 9.5 μL sterilized distilled water. Each group contained three samples, and each PCR was carried out in triplicate. The PCR protocol was as follows: 50°C for 2 min; 95°C for 10 min; 45 cycles of 95°C for 15 s, 60°C for 1 min; and a melting curve was performed starting at 65 up to 95°C with an increase of 0.5°C per 1 s to verify primer specificities. Expression levels of each gene was normalized to the GAPDH in the same samples. The primers were listed in Table 1.

**Table 1:** Real-time PCR primers.

**Gene symbol**	**Primers (5′–3′)**	**Gene symbol**	**Primers (5′–3′)**
Hes1-F	CCAGCCAGTGTCAACACGA	Nek2-F	TTCCATCCTCAGCCATGAAGA
Hes1-R	AATGCCGGGAGCTATCTTTCT	Nek2-R	CCTGCACTTGGACTTGGCAA
Hes5-F	AGTCCCAAGGAGAAAAACCGA	Sfn-F	GTGTGTGCGACACCGTACT
Hes5-R	GCTGTGTTTCAGGTAGCTGAC	Sfn-R	CTCGGCTAGGTAGCGGTAG
Hey1-F	GCGCGGACGAGAATGGAAA	Stmn1-F	TCTGTCCCCGATTTCCCCC
Hey1-R	TCAGGTGATCCACAGTCATCTG	Stmn1-R	AGCTGCTTCAAGACTTCCGC
HeyL-F	CAGCCCTTCGCAGATGCAA	Notch4-F	CTCTTGCCACTCAATTTCCCT
HeyL-R	CCAATCGTCGCAATTCAGAAAG	Notch4-R	TTGCAGAGTTGGGTATCCCTG
Id1-F	CCTAGCTGTTCGCTGAAGGC	Bmpr2-F	TTGGGATAGGTGAGAGTCGAAT
Id1-R	CTCCGACAGACCAAGTACCAC	Bmpr2-R	TGTTTCACAAGATTGATGTCCCC
Id2-F	ATGAAAGCCTTCAGTCCGGTG	Wnt7a-F	GGCTTCTCTTCGGTGGTAGC
Id2-R	AGCAGACTCATCGGGTCGT	Wnt7a-R	TGAAACTGACACTCGTCCAGG
Id3-F	GACGACATGAACCACTGCTAC	Fzd7-F	GCCACACGAACCAAGAGGAC
Id3-R	CCTGGCTAAGCTGAGTGCC	Fzd7-R	CGGGTGCGTACATAGAGCATAA
Cdkn1a-F	CCTGGTGATGTCCGACCTG	Sfrp1-F	CAACGTGGGCTACAAGAAGAT
Cdkn1a-R	CCATGAGCGCATCGCAATC	Sfrp1-R	GGCCAGTAGAAGCCGAAGAAC
Mdm2-F	TGTCTGTGTCTACCGAGGGTG	Ctnnbip1-F	GCCACAGCACTCCATCGAC
Mdm2-R	TCCAACGGACTTTAACAACTTCA	Ctnnbip1-R	GTCTCCGATCTGGAAAACGC
Tfdp1-F	TTGAAGCCAACGGAGAACTAAAG	Mapk10-F	AAGCCAGGGATTTGTTGTCTAAG
Tfdp1-R	TGGACTGTCCGAAGGTTTTTG	Mapk10-R	GGATGGAGGGAGACTCTCACT
Wee1-F	GTCGCCCGTCAAATCACCTT	Dkk2-F	CTGATGCGGGTCAAGGATTCA
Wee1-R	GAGCCGGAATCAATAACTCGC	Dkk2-R	CTCCCCTCCTAGAGAGGACTT
Ccne2-F	ATGTCAAGACGCAGCCGTTTA	Wwtr1-F	CATGGCGGAAAAAGATCCTCC
Ccne2-R	GCTGATTCCTCCAGACAGTACA	Wwtr1-R	GTCGGTCACGTCATAGGACTG
Gadd45g-F	GGGAAAGCACTGCACGAACT	Ppp2r2b-F	TGCCTTATATCTTCAGACCTCCA
Gadd45g-R	AGCACGCAAAAGGTCACATTG	Ppp2r2b-R	AATGTCAGCTTCAGTATGGCAG

### Immunostaining and image acquisition

Neomycin-damaged and undamaged cochleae were fixed in 4% PFA for 1 h at room temperature, washed with PBS, blocked with blocking solution (5% donkey serum, 0.5% Triton X100, 0.02% sodium azide, and 1% bovine serum albumin in pH 7.4 PBS) for 1 h at room temperature and then incubated with primary antibodies diluted in PBT1 (2.5% donkey serum, 0.1% Triton X100, 0.02% sodium azide, and 1% bovine serum albumin in pH 7.4 PBS) at 4°C for overnight. This was followed by washing with 0.1% (v/v) Triton X100 in pH 7.4 PBS three times and incubating with fluorescence-conjugated secondary antibody for 1 h at room temperature. After washing with 0.1% (v/v) Triton X100 in pH 7.4 PBS three times, the cochleae were mounted in antifade fluorescence mounting medium (DAKO). Anti-Myosin7a (Proteus Bioscience, #25-6790, 1:1,000 diluted in PBT1) and anti-Sox2 (Santa Cruz Biotechnology, #17320, 1:400 diluted in PBT1) primary antibodies were used. Donkey anti-rabbit Alexa Fluor 555 and 647 fluorescence-conjugated secondary antibodies (Invitrogen, #A-31572, #A-31573) were used for Myo7a, and donkey anti-goat Alexa Fluor 647 fluorescence-conjugated secondary antibody (Invitrogen, #A-21447) was used for Sox2. All the fluorescent secondary antibodies were diluted 1:400 in PBT2 (0.1% Triton X100 and 1% bovine serum albumin in pH 7.4 PBS). The Click-it EdU imaging kit (Invitrogen) was used after blocking to measure cell proliferation. The fluorescence images were obtained with a Zeiss LSM 710 confocal microscope and were analyzed using ImageJ (NIH) and Photoshop CS5 (Adobe Systems).

### RNA extraction for RNA-seq

Approximately 20,000 NLPs and 20,000 ULPs were used to extract total RNA with an RNeasy micro kit (QIAGEN). The RNA samples from NLPs and ULPs were split into three fractions for separate replicates.

### RNA-seq

The double-strand cDNA was synthesized from the total RNA obtained from the NLPs and ULPs using a TruSeq® RNA LT Sample Prep Kit v2 (Illumina). Illumina adapters were ligated to the cDNA molecules after end repair. The ligated cDNA was cleaned up with AmpureBeads (Beckman). The library was amplified using 10 cycles of PCR for the enrichment of adapter-ligated fragments. Transcriptome sequencing was carried out with the Illumina-Hiseq2500 system (Illumina).

### RNA-seq data analysis

The TopHat (version 1.3.2) and CuffLinks (version 2.2.1) pipeline was used for the alignment and gene expression counting of the RNA-seq data. The reference genome was mm9. The FPKM (Fragments per kilobase of exon per million fragments mapped) values of all mouse genes were summarized together for all samples (three NLP samples and three ULP sample). Spearman's rank correlation was calculated for all pair-wise combinations of samples based on the FPKM values of all mouse genes. The correlation plot was generated with the corrplot package in R. A total of 46,983 mouse RefSeq transcripts were included in the RNA-seq data, and the means and standard deviations of the normalized data were calculated. A value of *p* < 0.05 was considered statistically significant. The expression levels of all of the transcriptional units were measured according to their FPKM values, and a cutoff level of 0.1 was chosen as the lowest gene expression level. The Gene Ontology (GO) analysis was done with DAVID GO Annotation. The protein-protein interaction information was extracted from the STRING database. The interaction network graph was drawn by Cytoscope 3.4. Important gene lists of different signaling pathways (Wnt, Notch, TGFβ, Hippo) were determined based on information from the KEGG database.

### Statistical analysis

For each condition, at least three individual experiments were conducted. Data are presented as mean ± standard errors of the means (SEM), and GraphPad Prism6 software was used to analyze the data. Statistical significance was determined using a two-tailed, unpaired Student's *t*-test. A value of *p* < 0.05 was considered statistically significant.

## Results

### Neomycin injury significantly increases the HC regeneration ability of Lgr5+ progenitors

**Figure 1:**
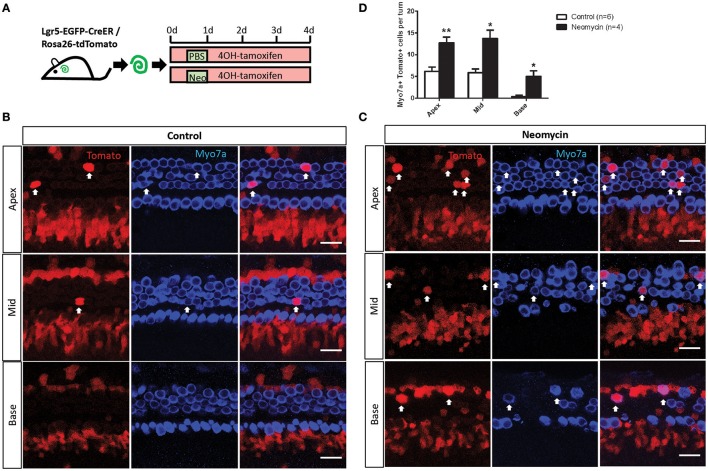
*In vitro* lineage tracing of Lgr5+ cells in the neomycin-treated and untreated cochleae of postnatal mice. **(A)** 4OH-tamoxifen was added to the culture medium of P1 Lgr5-EGFP-creER/Rosa26-tdTomato mouse cochleae throughout the entire culture period. Neomycin was added at 12 h after the start of the culture and was allowed to incubate for 12 h, and the same amount of PBS was added to the untreated cochlear culture medium for the same amount of time. The cochleae were examined after 4 days of culture. **(B,C)** Images of the neomycin-treated and untreated cochleae show that tdTomato+/Myo7a+ cells were found in the outer hair cell subset (arrow) in the apical, middle, and basal turns. **(D)** The cochleae were divided into three equal parts by length (apex, middle, and base), and all of the tdTomato+/Myo7a+ cells in each turn of the neomycin-treated cochleae and untreated cochleae were counted and statistically analyzed. ^*^p < 0.05, ^**^p < 0.01, *n* is shown in parentheses. Scale bars are 20 μm in **(B,C)**.

Lgr5+ progenitors can generate HCs in the neonatal mouse cochlea both *in vivo* and *in vitro* (Madisen et al., 2010; Chai et al., 2012). Here we performed an *in vitro* lineage-tracing experiment by crossing Lgr5-EGFP-creER mice with the Rosa26-tdTomato reporter strain (Pannier et al., 2009). P1 Lgr5-EGFP-creER/Rosa26-tdTomato double-positive mouse cochleae were dissected out and cultured in full medium with 500 nM 4OH-tamoxifen to lineage trace the Lgr5+ progenitors. The cochleae were damaged by neomycin as described in the Section Materials and Methods (Figure 1A). We found that significantly more tdTomato/Myo7a double-positive HCs were generated from NLPs compared to ULPs in all three turns of the cochlea (Figures 1B–D, *p* < 0.05, *n* ≧ 4), suggesting that the Lgr5+ progenitors generated significantly more HCs after neomycin injury *in vitro*.

### Neomycin injury increases the proliferation of Lgr5+ progenitors, but not significantly

**Figure 2:**
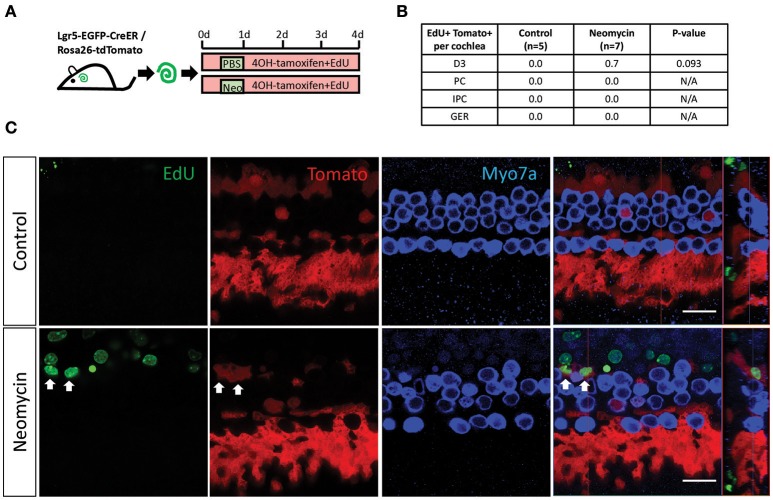
EdU labeling measures the proliferation of Lgr5+ cells in the neomycin-treated and untreated cochleae of postnatal mice. **(A)** 4OH-tamoxifen and EdU were added to the culture medium of P1 Lgr5-EGFP-creER/Rosa26-tdTomato mouse cochleae throughout the culture period. Neomycin was added at 12 h after the start of the culture and was allowed to incubate for 12 h, and the same amount of PBS was added to the untreated cochleae culture medium for the same amount of time. The cochleae were examined after 4 days of culture. **(B)** All of the tdTomato+/EdU+ cells found in the neomycin-treated cochleae and the untreated cochleae were counted and statistically analyzed. The *p* = 0.093, *n* is shown in parentheses. D3, the third-row Deiters' cells; PC, inner pillar cells; IPC, inner phalangeal cells; GER, the lateral greater epithelial ridge. **(C)** Images of the neomycin-treated and untreated cochleae show that tdTomato+/EdU+ cells were found in the neomycin-treated cochleae (arrows). Scale bars are 20 μm in **(B)**.

To determine the capacity of Lgr5+ progenitors in the damaged and undamaged cochleae to mitotically regenerate HCs, EdU was added to the culture medium from day 0 to day 4 of the culture (Figure 2A). Consistent with previous reports, there were no tdTomato+/EdU+ cells in the undamaged cochleae (Figures 2B,C). In contrast, tdTomato+/EdU+ cells, which represent the mitotically proliferated Lgr5+ progenitors, could be found in the damaged cochleae (Figure 2C), indicating that neomycin treatment induced the proliferation of Lgr5+ progenitors. However, due to the very small number of tdTomato+/EdU+ cells in neomycin-treated cochleae, the increase was not significant compared to the control group (Figure 2B, *p* = 0.093, *n* ≧ 5).

### Analysis of RNA-seq results

**Figure 3:**
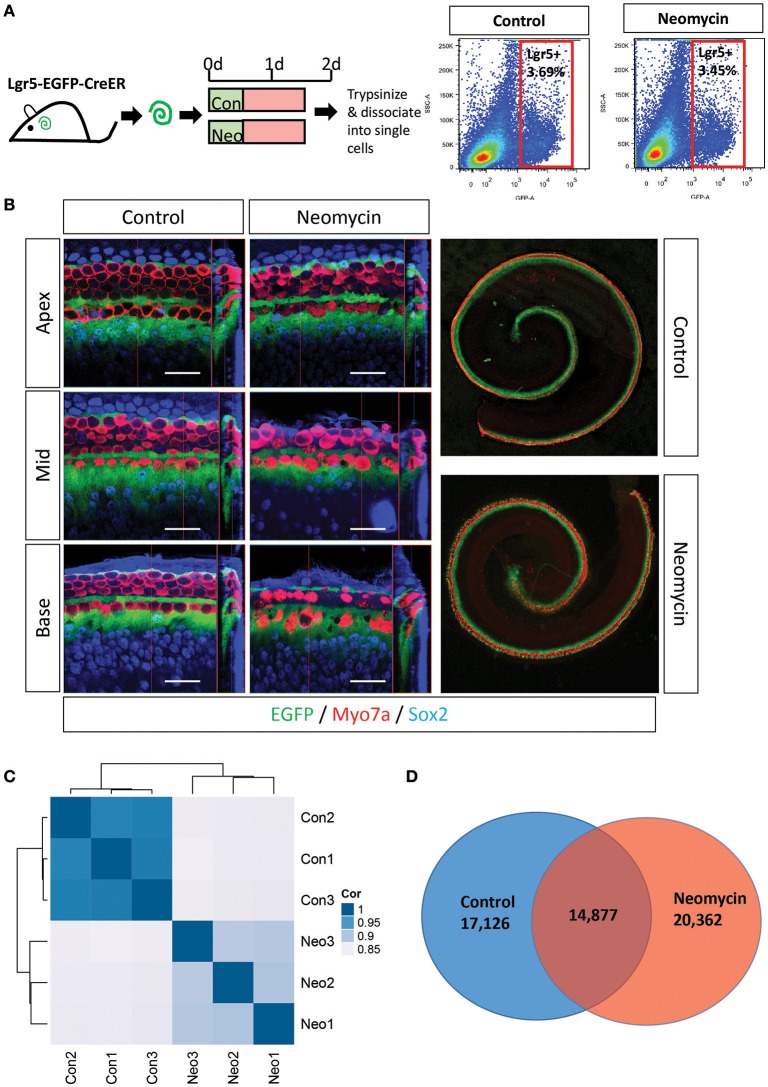
FACS sorting of Lgr5+ cells in the neomycin-treated and untreated cochleae of postnatal mice. **(A,B)** Neomycin was added for 12 h to the P1 Lgr5-EGFP-creER/Rosa26-tdTomato mouse cochleae, and the same amount of PBS was added to the untreated cochlear culture medium for the same amount of time. After a 24 h recovery period, the cochleae were trypsinized and dissociated into single cells for FACS sorting. **(C)** PCA analysis for all three replicates of NLPs (Neo1, Neo2, Neo3) and ULPs (Con1, Con2, Con3). **(D)** Venn diagram showing the number of genes expressed in NLPs (Neomycin) and ULPs (Control).

P1 Lgr5-EGFP-creER mice were sacrificed, and their cochleae were dissected out, cultured in full medium for 12 h, and then treated with 0.5 mM neomycin for 12 h to damage the HCs. The cochleae were allowed to recover for another 24 h before trypsinization and cell sorting (Figures 3A,B). After cell sorting, 20,000 isolated NLPs and 20,000 ULPs were collected and RNA-seq analysis was performed to determine their gene-expression profiles (supplementary Data Sheet 1). Principal component analysis was performed to assess the reproducibility of the measurements, and the NLP and ULP groups were well-separated by principal component 1 (Figure 3C). After excluding FPKM values below 0.1, 20,362 and 17,123 transcripts were examined separately in the NLPs and ULPs, respectively, and 14,877 transcripts were expressed in both cell populations (Figure 3D).

### Genes enriched in Lgr5+ progenitors from neomycin-damaged and undamaged cochleae

**Figure 4:**
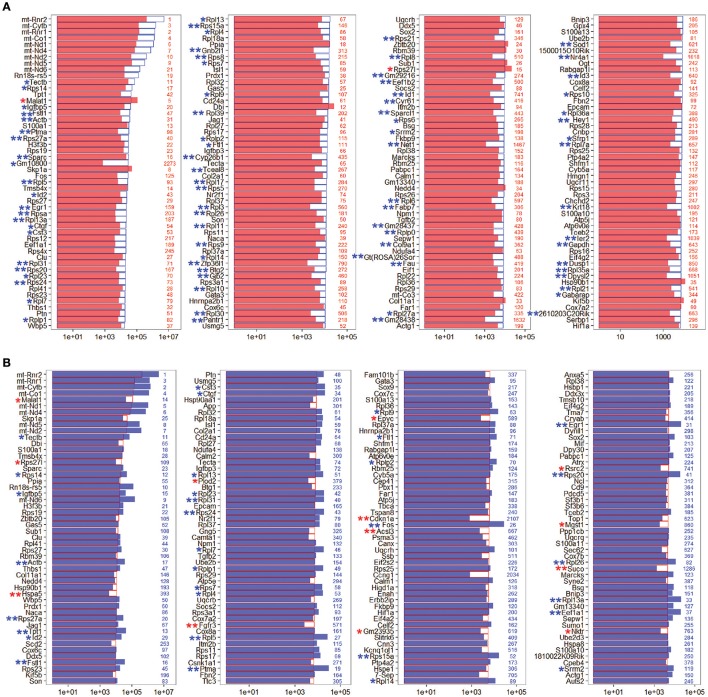
Expression levels of the top 200 genes in NLPs and ULPs. **(A)** Expression levels of the top 200 genes in ULPs in descending order. The red numbers on the right side of each panel represent the ranking of the same genes in NLPs. **(B)** Expression levels of the top 200 genes in NLPs in descending order. The blue numbers on the right side of each panel represent the ranking of the same genes in ULPs. ^*^p < 0.05, ^**^p < 0.01.

To determine the expression profiles of the richly expressed genes in NLPs and ULPs, the expression levels and abundance rankings of the most abundantly expressed genes were analyzed. Figure 4A shows the expression levels for the top 200 most abundant transcripts in ULPs (blue bars). The expression levels (red bars) and the abundance rankings (red numbers) of the same transcripts in NLPs are also illustrated for comparison. Similarly, Figure 4B shows the 200 most abundant transcripts in NLPs (red bars) compared to expression levels (blue bars) and abundance rankings (blue numbers) of the same transcripts in ULPs. As shown in both figures, most of the transcripts that were abundantly expressed in NLPs were also abundantly expressed in ULPs. However, *Gm10800, Net1, Gm28438, Nr4a1, Krt18, Ler2*, and *Dpysl2* (NLP rank > 1,000) were only richly expressed in ULPs, and *Cdkn1a, Ccng1*, and *Suco* (ULP rank > 1,000) were only richly expressed in NLPs.

### Differentially expressed genes in Lgr5+ progenitors from neomycin-damaged and undamaged cochleae

**Figure 5:**
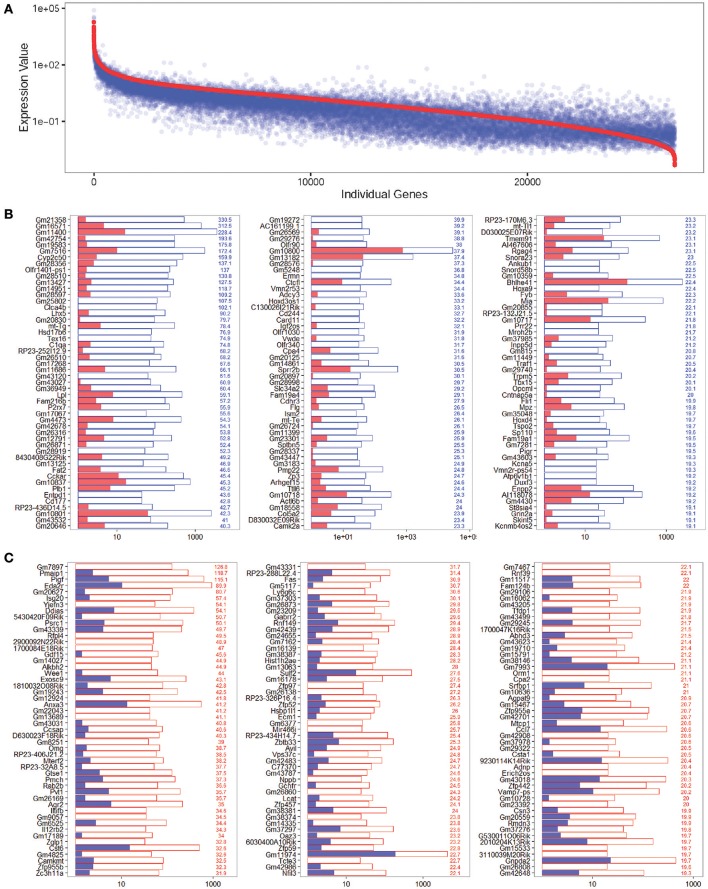
Differentially expressed genes in NLPs and ULPs. **(A)** All differentially expressed genes in NLPs and ULPs. The red line represents the expression level of transcripts from NLPs, and each blue dot represents the expression level of the same transcript from ULPs. **(B)** The 150 most differentially expressed genes in ULPs. The blue numbers on the right side of each panel represent the gene expression fold change in ULPs compared to NLPs. **(C)** The 150 most differentially expressed genes in NLPs. The red numbers on the right side of each panel represent the gene expression fold change in NLPs compared to ULPs.

In order to characterize the genes that are significantly differentially expressed in NLPs and ULPs, we selected the differentially expressed genes in NLPs and ULPs by comparing their expression levels (fold change > 2.0, *p* < 0.05). Figure 5A shows an overall picture of the expressed transcripts in NLPs and ULPs. We found 549 genes that were significantly upregulated and 1,817 genes that were significantly downregulated in the NLPs. Figures 5B,C show the top 150 differentially expressed genes in ULPs and NLPs. Among these differentially expressed genes, the functions of some genes have been reported previously. *Fgfr3* (Bermingham-McDonogh et al., 2001; White et al., 2012), *Egfr* (Saleem and Siddiqui, 2015), *Frem2* (Nadol et al., 2015), *Alms1* (Oshima et al., 2007b; Jagger et al., 2011), and *Lif* (Marzella et al., 1999; Su et al., 2015) were upregulated in NLPs, while *Hes1, Hes5* (Zheng et al., 2000; Zine et al., 2001; Li et al., 2008; Murata et al., 2009; Abdolazimi et al., 2016), *Hey1* (Tateya et al., 2011; Korrapati et al., 2013; Benito-Gonzalez and Doetzlhofer, 2014; Petrovic et al., 2015), *HeyL* (Kamaid et al., 2010), *Id1, Id2*, and *Id3* (Ozeki et al., 2005; Jones et al., 2006; Laine et al., 2010) were downregulated in NLPs. We did not find any functional reports for the other differentially expressed genes in the cochleae, and these should be further studied in the future.

### Cell cycle analysis

**Figure 6:**
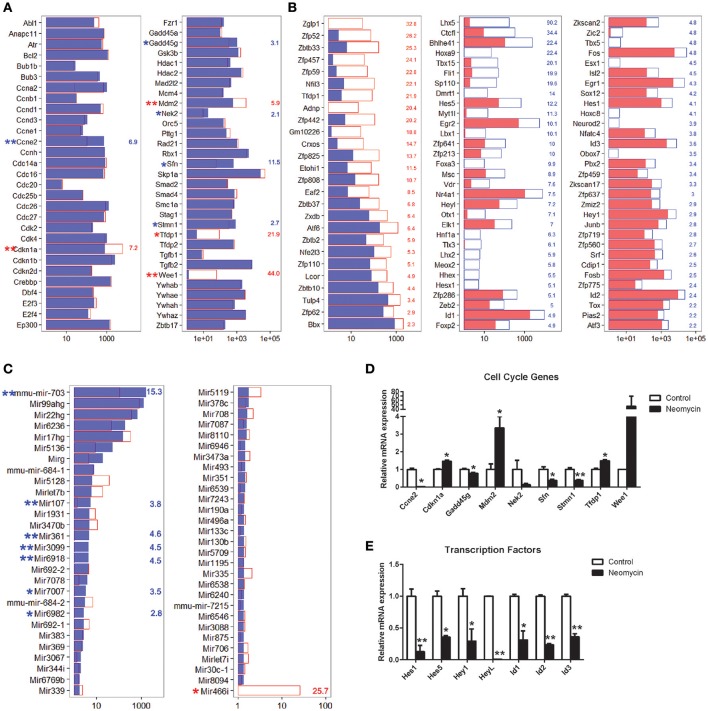
Expression of cell cycle-related genes, TFs, and miRNAs. **(A)** Expression levels of 60 genes that are involved in cell cycle regulation. **(B)** Fold change expression levels of differentially expressed transcription factors in descending order. **(C)** Expression levels of 57 microRNAs expressed in the cochlea. In all three panels, the red and blue numbers separately represent the fold change in upregulated and downregulated gene expression in NLPs compared to ULPs. ^*^p < 0.05, ^**^p < 0.01. **(D)** Real-time PCR analysis of the cell cycle genes. **(E)** Real-time PCR analysis of the TFs reported in the inner ear. ^*^p < 0.05, ^**^p < 0.01. *n* = 3.

Cells in the postnatal mammalian cochlea have exited the cell cycle, and they have very limited capacity for proliferation. In order to promote mitotic HC regeneration, it is important to induce HC progenitors to re-enter the cell cycle and to mitotically regenerate HCs. In the present study, we have demonstrated that neomycin injury could induce the proliferation of Lgr5+ progenitors; however, the detailed mechanism behind this proliferative ability remains unclear. It has been reported that some of the cell cycle genes play important roles in the cochlea. To identify the possible genes regulating the cell cycling of Lgr5+ progenitors, we examined the expression levels of cell cycle genes in NLPs and ULPs. We found that *Cdkn1a, Mdm2, Tfdp1*, and *Wee1* were significantly upregulated in NLPs and that *Ccne2, Gadd45g, Nek2, Sfn*, and *Stmn1* were significantly downregulated in ULPs (Figure 6A). Real-time PCR was also performed to confirm the RNA-seq results, and these two results were consistent (Figure 6D). Only the roles of *Cdkn1a* (Laine et al., 2007; Laos et al., 2017) and *Mdm2* (Mahmoodian Sani et al., 2016) in the inner ear have been described, and there are no reports of the roles of the other cell cycle genes we identified in ULPs and NLPs.

### Transcription factor analysis

Transcription factors (TFs) are able to bind to enhancer or promoter regions of their downstream target genes and control their expression levels. There are many TFs involved in inner ear development and HC regeneration. In the present study, we have demonstrated that NLPs have significantly greater HC regeneration capacity compared to ULPs (Figure 1, *p* < 0.05, *n* ≧ 4). However, the roles of a large number of TFs in the inner ear and in HC regeneration are unknown. To determine the TFs that might be involved in HC regeneration from Lgr5+ progenitors, we compared the expression levels of TFs in the mouse genome between NLPs and ULPs. Figure 6B shows the 88 significantly differentially expressed TFs in NLPs and ULPs (fold change > 2, *p* < 0.05). Some of the TFs that were downregulated in NLPs, including *Hes1, Hes5* (Zheng et al., 2000; Li et al., 2008; Murata et al., 2009; Abdolazimi et al., 2016), *Hey1* (Tateya et al., 2011; Korrapati et al., 2013; Benito-Gonzalez and Doetzlhofer, 2014; Petrovic et al., 2015), *HeyL* (Kamaid et al., 2010), *Id1, Id2*, and *Id3* (Ozeki et al., 2005; Jones et al., 2006; Laine et al., 2010), have been reported to play roles in negatively regulating HC fate and patterning regulation during inner ear development (Figure 6B). Real-time PCR was also performed to confirm the RNA-seq results, and these two results were consistent (Figure 6E). However, a significant number of the differentially expressed TFs have not been characterized in the inner ear before and need to be further studied in the future.

### MicroRNA analysis

MicroRNAs (miRNAs) are untranslated RNAs that control gene expression by binding to target mRNAs. A few miRNAs have been reported to play important roles in HC protection and HC regeneration (Jen et al., 1997; Li et al., 2010; Wang et al., 2010; Patel and Hu, 2012). We found that 149 miRNAs were uniquely expressed in ULPs, 151 miRNAs were uniquely expressed in NLPs, and 59 miRNAs were expressed in both ULPs and NLPs. Among these miRNAs, eight miRNAs were significantly differentially expressed in NLPs and ULPs (*p* < 0.05, fold change > 2; Figure 6C). *Mir466i* was upregulated in NLPs, while *Mir7007, mmu-mir-703, Mir107, Mir361, Mir6918, Mir6982*, and *Mir3099* were downregulated in NLPs. These miRNAs have not been characterized in the inner ear and need to be further studied in the future.

### Signaling pathway analysis

**Figure 7:**
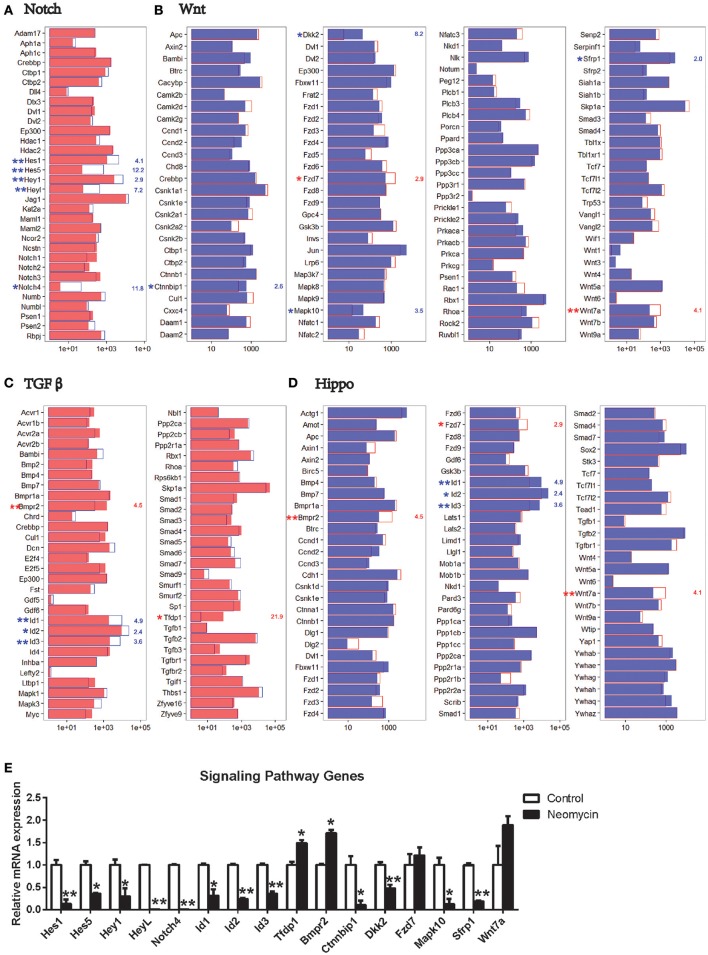
Expression of genes in the Notch, Wnt, TGFβ, and Hippo signaling pathways. **(A)** Expression levels of 32 genes that are important for the Notch signaling pathway. **(B)** Expression levels of 105 genes that are important for the Wnt signaling pathway. **(C)** Expression levels of 59 genes that are important for the TGFβ signaling pathway. **(D)** Expression levels of 80 genes that are important for the Hippo signaling pathway. In all four panels, the red and blue numbers separately represent the fold change in upregulated and downregulated gene expression in NLPs compared to ULPs. ^*^p < 0.05, ^**^p < 0.01. **(E)** Real-time PCR analysis of the signaling pathway genes. ^*^p < 0.05, ^**^p < 0.01. *n* = 3.

A few signaling pathways have been shown to be involved in inner ear development and HC regeneration. To determine which pathways might be involved in regulating HC regeneration from Lgr5+ progenitors, we compared the expression of genes involved in these pathways between the NLPs and ULPs. The most significantly different expression was in genes involved in the Notch and TGFβ pathways. Among the Notch signaling genes examined here, *Hes1, Hes5, Hey1, HeyL*, and *Notch4* were all significantly downregulated in NLPs compared to ULPs (Figure 7A). Among the TGFβ pathway genes, *Tfdp1* and *Bmpr2* were upregulated, while *Id1, Id2*, and *Id3* were downregulated in NLPs (Figure 7C). Among the Wnt pathway genes, *Wnt7a* and *Fzd7* were upregulated, while *Sfrp1, Ctnnbip1, Mapk10*, and *Dkk2* were downregulated in NLPs (Figure 7B). Among the Hippo pathway genes, *Bmpr2, Wnt7a*, and *Fzd7* were upregulated, while *Id1, Id2*, and *Id3* were downregulated in NLPs (Figure 7D). Real-time PCR was also performed to confirm the RNA-seq results, and these two results were consistent (Figure 7E). The differential expression of genes in the Notch, TGFβ, Wnt, and Hippo pathways suggests that these pathways might be involved in neomycin-induced HC regeneration. Some studies have shown that the Notch and Wnt pathways regulate the development of inner ear progenitor cells (Chai et al., 2012; Kelly et al., 2012). Thus, although the TGFβ and Hippo pathways are not well-studied they are probably the pathways that regulate HC regeneration.

### Gene ontology and network analysis of the genes that are differentially expressed in Lgr5+ cells from neomycin-damaged and undamaged cochleae

**Figure 8:**
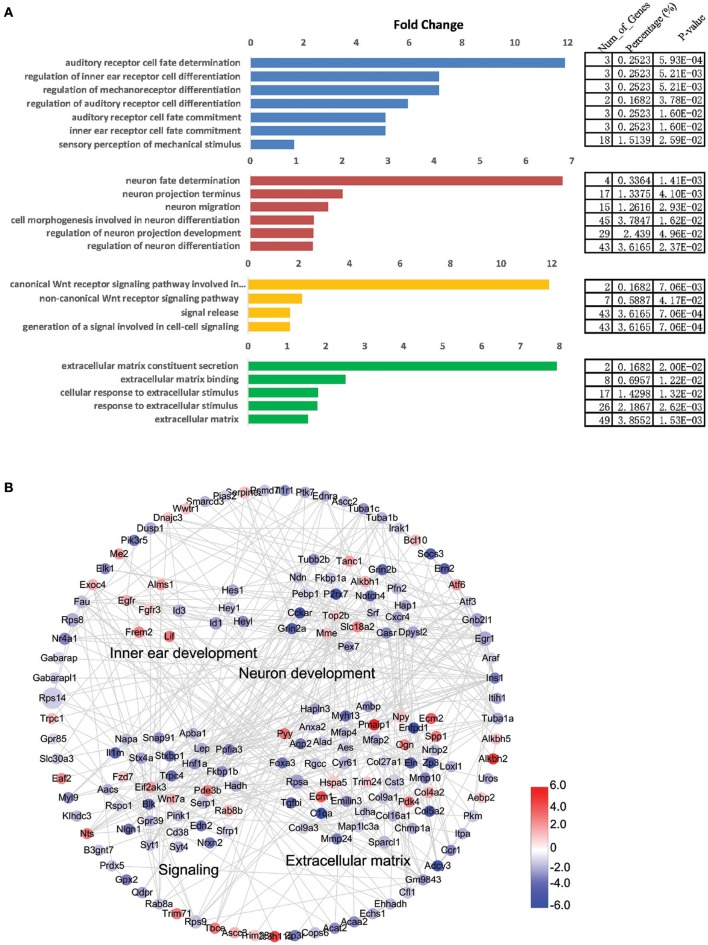
Gene ontology (GO) and network analysis of the differentially expressed genes in NLPs and ULPs. **(A)** GO analysis of differentially expressed genes in NLPs and ULPs. **(B)** STRING protein-protein interaction analysis of genes that are upregulated (red) and downregulated (blue) in NLPs. The gray lines indicate protein-protein interactions in the STRING database. The DAVID GO annotation was used to cluster the genes according to biological function.

To view the interactions and connections of genes that are differentially expressed in NLPs and ULPs, we constructed a STRING protein-protein interaction network for the significantly differentially expressed genes (fold change > 2.0, *p* < 0.05) with the functional categories in the gene ontology (GO) analysis (DAVID; Figure 8B). This comprehensive analysis revealed a complex gene network that might regulate HC regeneration. We also applied GO analysis to genes with altered expression levels in NLPs (fold change > 2.0, *p* < 0.05; Figure 8A). The genes with altered expression in NLPs were highly enriched in functional categories such as auditory receptor cell fate determination, neuron fate determination, signaling, and extracellular matrix formation and maintenance.

## Discussion

The Lgr5+ cells of the cochlea are reported to be an enriched population of HC progenitors that have high potential for HC regeneration. Previous studies have shown that Lgr5+ progenitors regenerate more HCs upon damage *in vitro* and *in vivo*, but the detailed mechanisms behind NLP HC regeneration and the gene expression profile differences between NLPs and ULPs are not well-understood. Here, we found that NLPs show much greater capacity for HC regeneration than ULPs and that NLPs have slightly greater proliferation than ULPs. We carried out RNA-seq experiments to analyse the detailed gene expression profile of NLPs and ULPs. We first analyzed the top 200 most abundant genes and top 150 most differentially expressed genes in NLPs and ULPs, and we found 9 cell cycle genes, 88 TFs, and 16 signaling pathway genes that were differentially expressed in NLPs and ULPs. Some of the differentially expressed genes have been reported to be involved in inner ear development and HC regeneration in neonatal mice. However, many of the other genes, which might be potential targets regulating HC regeneration, have not been well-studied and need to be further studied in the future.

### Differentially expressed genes in NLPs and ULPs

We have demonstrated that NLPs are able to regenerate many more HCs than ULPs, which has been reported previously (Bramhall et al., 2014). To determine the detailed mechanisms behind this difference, we compared the expression levels of all of the transcripts in NLPs with those of ULPs. We identified 549 genes that were significantly upregulated and 1,817 genes that were significantly downregulated in the NLPs compared to the ULPs. The functions of some of the differentially expressed genes have been reported previously. *Egfr* governs the regenerative proliferation of auditory p75+ SCs in birds and mammals after HC damage (Saleem and Siddiqui, 2015). Mutation of *Fgfr3* causes hearing loss and inner ear defects and might be involved in regulating the proliferation of SCs (Bermingham-McDonogh et al., 2001; White et al., 2012). Mutations in *Frem2* have been linked to Fraser's syndrome, which is a rare autosomal recessive disorder with a spectrum of malformations, including malformations of the ear (Nadol et al., 2015). Mutations in *Alms1* cause Alstrom's syndrome, which is an autosomal recessive syndromic genetic disorder with sensorineural hearing loss (Bermingham-McDonogh et al., 2001; White et al., 2012). *Lif* controls neural differentiation and maintenance of stem cell-derived murine spiral ganglion neuron precursors (Marzella et al., 1999; Su et al., 2015). *Hes1, Hes5, Hey1*, and *HeyL* are downstream effectors of the Notch pathway and have been reported to negatively regulate HC differentiation and regeneration (Zheng et al., 2000; Zine et al., 2001; Li et al., 2008; Murata et al., 2009; Abdolazimi et al., 2016). *Id1, Id2*, and *Id3* are downstream targets of the TGFβ and Hippo pathways and regulate HC formation during inner ear development (Ozeki et al., 2005; Jones et al., 2006; Laine et al., 2010; Zhan et al., 2017). These results support our hypothesis that NLPs have a much greater potential to generate HCs in the neonatal cochlea than ULPs. However, it should be noted that not all of the differentially expressed genes that we identified have been characterized, so there might still be mechanisms at work that we are not yet aware of.

### Cell cycle analysis

Mammalian cochlear SCs do not enter the cell cycle or proliferate after birth under normal circumstances. We demonstrated that Lgr5+ progenitors that re-enter into cell cycle and proliferate could be found in the neomycin-damaged cochlea, but no such cells could be found in the control group. To identify the possible genes regulating the cell cycling of Lgr5+ progenitors, we compared cell cycle gene expression in NLPs and ULPs. *Tfdp1* (Vairapandi et al., 2002; Yasui et al., 2003; Lu et al., 2016), which was upregulated in NLPs, is a positive regulator of the cell cycle, while *Gadd45g* and *Sfn*, which were downregulated in NLPs, are negative regulators of the cell cycle (Liu et al., 2010; Aktary et al., 2013; Vogel and Herzinger, 2013; Phan et al., 2015). However, *Cdkn1a* (Duan et al., 2005; Laine et al., 2007; Mollapour et al., 2010; Laos et al., 2017), Wee1 (Lin et al., 2006; Tominaga et al., 2006; De Schutter et al., 2007; Frum et al., 2009), and *Mdm2* (Helps et al., 2000; Giono and Manfredi, 2007; Shangary et al., 2008), which were upregulated in NLPs, have been reported to play roles in regulating cell proliferation, and *Nek2* (Schultz et al., 1994; Fry et al., 1995; Nabilsi et al., 2013; He et al., 2016), *Stmn1* (Johnsen et al., 2000; Wang et al., 2011; Li X. et al., 2015; Guo et al., 2016; Zhou et al., 2016), and *Ccne2* (Chen et al., 2015; Clausse et al., 2016; Gorjala et al., 2016), which were downregulated in NLPs, have been reported to negatively regulate cell proliferation. Interestingly, these genes (*Cdkn1a, Mdm2, Wee1, Nek2, Stmn1*, and *Ccne2*) are all involved in p53-dependent cell cycle arrest (Fry et al., 1995; Giono and Manfredi, 2007; Kiernan, 2013; Clausse et al., 2016; Zhou et al., 2016; Laos et al., 2017), and the changes in expression of these genes might be because neomycin injury also slightly activates the p53 pathway in Lgr5+ progenitor cells. The expression changes of *Tfdp1, Gadd45g*, and *Sfn* promote cell cycle progression, while the expression changes of *Cdkn1a, Mdm2, Wee1, Nek2, Stmn1*, and *Ccne2* repress cell cycle progression, which might be the reason for the lack of significant proliferation in the neomycin treated cochleae.

### Transcription factor analysis

TFs, which bind to the promoter region of their downstream target genes and regulate gene expression, are important factors involved in development, cell proliferation, differentiation, and other cellular functions. *Hes1, Hes5, Hey1*, and *HeyL* are downstream effectors of Notch signaling, which is a well-known signaling pathway regulating HC fate and patterning (Malgrange et al., 2002; Li et al., 2003; Saito et al., 2009; Hartman et al., 2010; Kamaid et al., 2010; Pan et al., 2010; Jeon et al., 2011), and inhibition of Notch induces significant HC regeneration in newborn mice (Li et al., 2003; Kamaid et al., 2010). *Id1, Id2*, and *Id3* (inhibitors of differentiation and DNA binding) regulate HC formation during development by negatively regulating Atoh1 (Ozeki et al., 2005; Jones et al., 2006; Laine et al., 2010; Zhan et al., 2017). These data support our hypothesis that these TFs participate in the increased HC regeneration of NLPs. Furthermore, we have identified many TFs that have not been characterized in the inner ear before. *Croxs* (Calderon et al., 2012), *Lcor* (Yu et al., 2014), *Nfil3* (Seillet et al., 2014a,b; Malishkevich et al., 2015), *Adnp* (Nakajima et al., 2008; Oz et al., 2012), and *Tfdp1* (Vairapandi et al., 2002; Yasui et al., 2003; Lu et al., 2016) were upregulated in NLPs, and these genes have all been shown previously to have a stimulatory effect on the cell cycle or on the growth of some tumor cells and some normally proliferative tissues and/or on neurodevelopment and lymphoid cell development. Some of the TFs that were downregulated in NLPs, including *Esx1* (Asanoma et al., 2015), *Bhlhe41* (Cui et al., 2016), and *Dmrt1* (Krentz et al., 2009; Zou et al., 2016), have been reported to play critical roles in negatively regulating cancer cell and stem cell growth in other tissues. The involvement of these genes in the differential HC regeneration capacity of NLPs and ULPs should be investigated in the future.

### MicroRNA analysis

miRNAs bind to target mRNAs and signal their degradation, and they play a key role in the control of gene expression and the regulation of cellular differentiation, proliferation, and apoptosis. Several miRNAs have been reported to play important roles in inner ear development (Jen et al., 1997; Li et al., 2010; Wang et al., 2010; Patel and Hu, 2012). We found eight significantly differentially expressed microRNAs in NLPs and ULPs (*p* < 0.05, fold change > 2). *Mir466i* was upregulated in NLPs, while *Mir7007, mmu-mir-703, Mir107, Mir361, Mir6918, Mir6982*, and *Mir3099* were downregulated in NLPs. Among these miRNAs, *Mir107* (Chen et al., 2013; Song et al., 2015; Xia et al., 2016; Yang et al., 2016) and *Mir361* (Wu et al., 2013; Jacques et al., 2014; Chen et al., 2016; Sun et al., 2016) have been reported to suppress tumor growth and stem cell growth. However, none of the eight miRNAs have been reported previously in the inner ear and need to be further studied in the future.

### Signaling pathway analysis

Several signaling pathways have been shown to be involved in inner ear development and HC regeneration (Malgrange et al., 2002; Yamamoto et al., 2006; Bermingham-McDonogh and Reh, 2011; Chai et al., 2012; Kelly et al., 2012). Among these signaling pathways, Wnt and Notch are the two most well-studied pathways in HC regeneration (Bermingham-McDonogh and Reh, 2011; Chai et al., 2012; Kelly et al., 2012). Overexpression of Wnt increases SC proliferation and Lgr5+ cell clustering and leads to increased numbers of EdU+/Lgr5-EGFP+ cells (Zhao et al., 2006; Madisen et al., 2010; Chai et al., 2012; Bohnenpoll et al., 2014). Inhibition of Notch significantly increases HC differentiation from SCs/Lgr5+ progenitors (Malgrange et al., 2002; Saito et al., 2009; Hartman et al., 2010; Pan et al., 2010; Jeon et al., 2011). Notch inhibition also increases HC regeneration through induction of the Wnt pathway (Li et al., 2003). Other pathways, such as Shh (Liu et al., 2002; Loh et al., 2014), Hippo (Murillo-Cuesta et al., 2015), and TGFβ (Kawamoto et al., 2003; Butts et al., 2005; Yang et al., 2009; McLean et al., 2017), also play important roles in inner ear development. In a recent report, a TGFβ receptor inhibitor increased Lgr5+ cell expansion *in vitro* (Du et al., 2013). To determine which pathways might be involved in regulating HC regeneration from Lgr5+ progenitors, we examined the differences in expression of pathway-related genes that might play a role in inner ear development between the NLPs and ULPs.

*Hes1, Hes5, Hey1, HeyL*, and *Notch4* are genes of the Notch signaling pathway, which is a well-known signaling pathway regulating HC fate and patterning (Zheng et al., 2000; Zine et al., 2001; Zine and de Ribaupierre, 2002; Li et al., 2008; Murata et al., 2009; Tateya et al., 2011; Korrapati et al., 2013; Ku et al., 2014; Petrovic et al., 2015; Abdolazimi et al., 2016), and were significantly downregulated in NLPs. Inhibition of Notch can lead to HC regeneration mainly by inducing SCs to transdifferentiate into HCs (Malgrange et al., 2002; Saito et al., 2009; Hartman et al., 2010; Pan et al., 2010; Jeon et al., 2011). Although, there is no direct evidence for regulating HC fate and patterning, *HeyL* is thought to be a target and potential Notch effector of Notch signaling (Kamaid et al., 2010; Bui et al., 2017). *Notch4* is involved in the migration and invasion of several kinds of cancers (Melchor et al., 2009; Qian et al., 2016).

Five genes of the TGFβ pathway were differentially expressed. *Tfdp1* and *Bmpr2* were upregulated, while *Id1, Id2*, and *Id3* were downregulated in NLPs. *Id1, Id2*, and *Id3* regulate HC formation during development by negatively regulating Atoh1 (Ozeki et al., 2005; Jones et al., 2006; Laine et al., 2010; Zhan et al., 2017). *Tfdp1* encodes a TF that binds to the promoter regions of a number of genes whose products are involved in cell cycle regulation or in tumor proliferation (Vairapandi et al., 2002; Yasui et al., 2003; Liu S. et al., 2016; Lu et al., 2016). *Bmpr2* encodes a member of the bone morphogenetic protein receptor family of transmembrane serine/threonine kinases that play important roles in stem cell differentiation (Zeng et al., 2012; Larabee et al., 2015; Ramos-Solano et al., 2015). The roles of these genes in HC regeneration remain unclear and need to be studied in the future.

Six genes of the Wnt pathway were differentially expressed. *Wnt7a* and *Fzd7* were upregulated, while *Sfrp1, Ctnnbip1, Mapk10*, and *Dkk2* were downregulated in NLPs. *Wnt7a*, a gene coding for one of the Wnt genes (Chiu et al., 2010; Qu et al., 2013; King et al., 2015; Qiu et al., 2016), and *Fzd7* (Sienknecht and Fekete, 2008; Yang et al., 2011; Song et al., 2014; Deng et al., 2015; Wang K. et al., 2015), one of the Wnt protein receptors, were both upregulated in NLPs and have been reported previously to be expressed in the inner ear (Wang K. et al., 2015). *Wnt7a* and *Fzd7* are both reported to induce cell proliferation and differentiation in other tissues and cell types (Sienknecht and Fekete, 2008; Chiu et al., 2010; Yang et al., 2011; Song et al., 2014; Deng et al., 2015; King et al., 2015; Qiu et al., 2016), but their roles in the inner ear remain unclear and need to be further studied in the future. *Sfrp1*, which codes for a secreted Wnt antagonist that directly interacts with Wnt ligand (Satoh et al., 2008; Lee et al., 2010; Tong et al., 2015), is downregulated in NLPs. *Ctnnbip1*, which is downregulated in NLPs, encodes a protein that negatively regulates Wnt signaling by preventing the interaction between β-catenin and TCF/LEF family members (Guo et al., 2015; Qi et al., 2015; Li and Luo, 2017). *Mapk10*, a target of miR-27a-3p, is envolved in nasopharyngeal carcinoma cell proliferation and migration (Phillips et al., 2011). *Dkk2*, which is downregulated in NLPs, encodes a protein that antagonizes canonical Wnt signaling by inhibiting LRP5/6 interactions with Wnt (Mukhopadhyay et al., 2006; Fleury et al., 2010).

Six genes of the Hippo pathway were differentially expressed. *Bmpr2, Fzd7*, and *Wnt7a* were upregulated in NLPs, while *Id1, Id2* and *Id3* were downregulated in NLPs. *Id1, Id2*, and *Id3*, as mentioned above, have been reported to regulate HC formation during inner ear development (Ozeki et al., 2005; Jones et al., 2006; Laine et al., 2010; Zhan et al., 2017). *Bmpr2*, as mentioned above, plays important roles in stem cell differentiation (Zeng et al., 2012; Larabee et al., 2015; Ramos-Solano et al., 2015). The roles of these genes and the Hippo pathway in HC regeneration remain unclear and need to be studied in the future.

### STRING prediction of inner ear HC development

We used the GO analysis to determine the functional categories of the differentially expressed genes in NLPs and ULPs, and the STRING database was used to construct a protein-protein interaction network for the differentially expressed genes. Importantly, NLPs and ULPs have very different expressions of genes involved in inner ear development, neuron differentiation, signaling pathways, and extracellular matrix. Among the genes involved in inner ear development, *Fgfr3* (Bermingham-McDonogh et al., 2001; White et al., 2012), *Egfr* (Saleem and Siddiqui, 2015), *Frem2* (Nadol et al., 2015), *Alms1* (Oshima et al., 2007b; Jagger et al., 2011), and *Lif* (Marzella et al., 1999; Su et al., 2015), which are all positively involved in inner ear development and HC differentiation, were upregulated in NLPs. *Hes1, Hes5* (Zheng et al., 2000; Zine et al., 2001; Li et al., 2008; Murata et al., 2009; Abdolazimi et al., 2016), *Hey1* (Tateya et al., 2011; Korrapati et al., 2013; Benito-Gonzalez and Doetzlhofer, 2014; Petrovic et al., 2015), *HeyL* (Kamaid et al., 2010), *Id1, Id2*, and *Id3* (Ozeki et al., 2005; Jones et al., 2006; Laine et al., 2010), which all negatively regulate inner ear development and HC differentiation, were downregulated in NLPs. It would be interesting to investigate the involvement of these genes in regulating HC regeneration of Lgr5+ progenitor cells in the future.

In summary, we found that NLPs have a greater capacity to regenerate HCs and a slightly greater capacity to proliferate compared to ULPs. We investigated the differences in the transcriptomes between the NLPs and ULPs, and we identified several differentially expressed genes that might regulate the ability of Lgr5+ progenitor cells to proliferate and to regenerate functional HCs. Lastly, to further analyze the interactions and connections of the differentially expressed genes in HC regeneration, we constructed a STRING protein-protein interaction network. The transcriptomes of the NLPs and ULPs reported here provide numerous target genes that should be characterized for HC regeneration in the future.

## Author contributions

SZ, HS, XG and RC designed the study. SZ, YZ, HZ, LG, XX, XCZ, and JQ performed the laboratory experiments. RC, SZ, PY, YZ, XLZ, XQ, FC, HS, XG, YH, and YL contributed to critical discussion and data analysis. SZ, MW, HS, and XG and RC wrote the paper. All authors read and approved the final manuscript.

### Conflict of interest statement

The authors declare that the research was conducted in the absence of any commercial or financial relationships that could be construed as a potential conflict of interest.
